# Antibiotic Prescribing Patterns for Pulpitis in Pediatric Dentistry: A Systematic Review and Meta-Analysis

**DOI:** 10.3390/antibiotics15060586

**Published:** 2026-06-08

**Authors:** Carmen Machuca-Portillo, Cira Suárez-Marchena, Lucy Chandler-Gutiérrez, María José Barra-Soto, Lydia López-del Valle, Juan J. Segura-Egea

**Affiliations:** 1 Department of Stomatology, Pediatric Dentistry Division, School of Dentistry, University of Sevilla, C/Avicena s/n, 41009 Sevilla, Spain; chandler@us.es (L.C.-G.); mbarra@us.es (M.J.B.-S.); 2 School of Dental Medicine, Medical Sciences Campus, University of Puerto Rico, UPR, San Juan Medical Center Building, San Juan 00936, Puerto Rico; lydia.lopez1@upr.edu; 3 Department of Stomatology, Endodontic Division, School of Dentistry, University of Sevilla, C/Avicena s/n, 41009 Sevilla, Spain; segurajj@us.es

**Keywords:** pediatric dentistry, antibiotic prescribing, pulpitis, antimicrobial stewardship, clinical guidelines

## Abstract

**Background**: Pulpitis is a common cause of dental pain in children and is primarily an inflammatory condition that can be effectively managed with local operative treatment. Although antibiotics are indicated only in cases of systemic involvement or infection spread, they are frequently overprescribed in dental practice. This misuse contributes to antimicrobial resistance and adverse health outcomes. This systematic review aimed to evaluate antibiotic prescribing practices for pulpitis in pediatric patients and to assess adherence to current clinical guidelines. **Methods**: A systematic review was conducted in accordance with the PRISMA 2020 statement and registered in PROSPERO (CRD420261342269). A comprehensive search was performed in PubMed/MEDLINE, Scopus, and Embase up to March 2026. Observational studies assessing antibiotic prescribing practices among pediatric dentists were included. A meta-analysis of proportions was conducted using a random-effects model. Risk of bias was assessed using the Joanna Briggs Institute Checklist, and certainty of evidence was evaluated using the GRADE approach. **Results**: Five cross-sectional studies were included. Antibiotic prescribing rates for pulpitis ranged from 0.6% to 50.0%. The pooled prevalence of antibiotic prescribing was 14.0% (95% CI: 5.0–33.5%), with high heterogeneity across studies (I^2^ = 95%). Amoxicillin and amoxicillin–clavulanic acid were the most commonly prescribed first-line antibiotics, while clindamycin was the most frequently reported alternative in patients with penicillin allergy. Treatment duration was generally consistent, ranging from 5 to 7 days. **Conclusions**: Although pediatric dentists tend to prescribe antibiotics more conservatively than general practitioners, inappropriate use remains prevalent, particularly in conditions such as pulpitis where antibiotic therapy is not indicated. These findings highlight a persistent gap between evidence-based recommendations and clinical practice and underscore the need for targeted antimicrobial stewardship strategies to optimize antibiotic use in pediatric dentistry.

## 1. Introduction

Dental pain is one of the most frequent reasons for seeking dental care in children and is commonly associated with pulpal pathology resulting from untreated dental caries, trauma, or restorative failure. Pulpitis, characterized by inflammation of the dental pulp, may initially present as a reversible condition but can progress to irreversible pulpitis and ultimately to pulp necrosis if left untreated [1]. Although pulpitis is primarily an inflammatory process, its progression may lead to apical periodontitis and, in advanced cases, to the spread of infection beyond the tooth.

The extension of odontogenic infections into surrounding tissues can result in potentially serious complications, including facial cellulitis and deep neck infections, particularly in pediatric patients with developing immune systems [2,3,4]. In such cases, especially when systemic involvement is present—manifested by fever, diffuse swelling, or lymphadenopathy—antibiotic therapy may be indicated as an adjunct to local operative treatment. However, in the absence of systemic signs or infection spread, most pulpal and periapical conditions, including pulpitis, can be effectively managed through local dental interventions alone [2,3,4,5].

Despite clear clinical recommendations, antibiotics continue to be prescribed inappropriately in dental practice, particularly for conditions such as pulpitis where their use is not indicated [5,6,7]. This pattern of overprescribing represents a significant concern, as unnecessary antibiotic exposure contributes to the development of antimicrobial resistance, a major global public health threat [8]. In addition to resistance, antibiotic use is associated with adverse effects ranging from mild gastrointestinal disturbances to severe allergic reactions, and emerging evidence suggests a potential link between early antibiotic exposure and long-term health outcomes in children [9,10,11].

Current guidelines from the American Academy of Pediatric Dentistry (AAPD) and other professional organizations clearly state that antibiotics are not indicated for localized odontogenic conditions such as pulpitis in the absence of systemic involvement [12,13]. Instead, definitive operative treatment, including pulpotomy, pulpectomy, or extraction, should be prioritized. Antibiotic therapy should be reserved for cases involving spreading infection or systemic compromise, where prompt medical management is required [14,15].

Although antibiotic prescribing in dentistry has been widely investigated, most studies have focused on odontogenic infections in general, general dental practitioners, or mixed populations. In contrast, evidence specifically addressing antibiotic prescribing for pulpitis in pediatric dentistry remains limited. Because pulpitis represents a localized inflammatory condition for which antibiotics are generally not indicated in the absence of systemic involvement, understanding prescribing behavior in this specific clinical scenario is particularly relevant for antimicrobial stewardship. This represents an important gap in the literature, as understanding prescribing behaviors in this context is essential for improving antimicrobial stewardship in pediatric dental care [16].

Therefore, the aim of this systematic review is to evaluate and quantify antibiotic prescribing practices for pulpitis in pediatric patients, and to assess their adherence to current clinical guidelines. By identifying patterns of use and discrepancies between evidence and practice, this review seeks to provide insights that may support the optimization of antibiotic prescribing and the implementation of targeted stewardship strategies in pediatric dentistry.

## 2. Materials and Methods

### 2.1. Protocol and Registration

This systematic review was conducted in accordance with the Preferred Reporting Items for Systematic Reviews and Meta-Analyses (PRISMA) 2020 statement [17]. The review protocol was prospectively registered in the International Prospective Register of Systematic Reviews (PROSPERO) under registration number CRD420261342269.

### 2.2. Review Question

The review question was formulated using the CoCoPop (condition, context, population) framework, which is appropriate for studies addressing prevalence and patterns of clinical practice [18].

Condition (Co): Antibiotic prescribing practices for pulpitis.

Context (Co): Clinical dental practice settings.

Population (Pop): Pediatric dentists managing children and adolescents.

Based on this framework, the research question was defined as “How do pediatric dentists prescribe antibiotics for pulpitis in children and adolescents, in terms of patterns, indications, and adherence to clinical guidelines?”

### 2.3. Eligibility Criteria

Eligibility criteria were defined a priori according to the CoCoPop framework.

Inclusion criteria:

Studies were included if they met all of the following criteria:

Study design: Observational studies (cross-sectional, case–control, prospective or retrospective cohort studies).

Population: Pediatric dentists treating pediatric patients.

Focus: Assessment of antibiotic prescribing practices for pulpitis or pulpal-related conditions.

Outcomes: Reporting data on prescribing frequency, patterns, or adherence to guidelines.

Publication period: Last 10 years.

Language: English.

Sample size: Minimum of 50 participants.

Exclusion criteria:

Studies were excluded if:

They involved only general dentists or mixed populations without separate data for pediatric dentists.

They focused exclusively on adult populations.

They assessed patient perspectives rather than clinician prescribing behavior.

They were clinical trials, systematic reviews, case reports, case series, editorials, or commentaries.

They did not specifically address pulpitis.

### 2.4. Search Strategy

A comprehensive literature search was conducted in PubMed/MEDLINE, Scopus, and Embase from January 2016 to March 2026. The final search was performed on 15 March 2026.

The search strategy combined controlled vocabulary (MeSH and Emtree terms) and free-text keywords related to antibiotic prescribing and pulpitis. The main search concepts included: “pulpitis”, “antibiotics” OR “antimicrobial agents”. Boolean operators (AND, OR) and truncation were applied as appropriate. The search strategy was adapted for each database according to its indexing system. No restrictions on study design were applied during the initial search. Filters for publication date and language were applied during the screening phase. The search strategy was intentionally focused on pulpitis and pulpal-related conditions, as the primary objective of this review was to evaluate antibiotic prescribing practices specifically in cases of pulpitis rather than in odontogenic infections in general.

The complete search strategies for each database are presented in Table 1.

### 2.5. Selection of Studies

Study selection was performed independently by three reviewers (C.M.-P., C.S.-M., and L.C.-G.). Prior to study selection and data extraction, the reviewers conducted calibration exercises using a sample of studies to ensure consistency and agreement regarding eligibility criteria and extracted variables. After removal of duplicates, titles and abstracts were screened to identify potentially eligible studies. Full-text articles were then assessed against the predefined eligibility criteria.

Disagreements were resolved through discussion and consensus. Additionally, the reference lists of included studies were manually screened to identify any further relevant publications.

### 2.6. Data Extraction

Data extraction was conducted independently by two reviewers (C.M.-P. and C.S.-M.) and verified by a third reviewer (J.J.S.-E.) to ensure accuracy and consistency. Discrepancies were resolved through consensus.

The following data were extracted: study characteristics (authors, year, country, study design); survey characteristics (number of questionnaires distributed, response rate); diagnostic criteria for pulpitis and related conditions; number of participating pediatric dentists; prevalence of antibiotic prescribing; first-line antibiotic choice; alternative antibiotics for penicillin-allergic patients; and duration of antibiotic therapy. Data were systematically organized in tabular format.

### 2.7. Data Analysis

A qualitative synthesis of the included studies was initially performed. When sufficient data were available, a meta-analysis of proportions was conducted to estimate the pooled prevalence of antibiotic prescribing among pediatric dentists for pulpitis, which was defined as the primary outcome.

The meta-analysis was performed using OpenMeta[Analyst] software (Brown University, version 10.10) [19], applying a DerSimonian–Laird random-effects model to account for between-study variability. Proportion data were logit-transformed prior to pooling in order to stabilize variance and better approximate normality assumptions. Pooled estimates and their corresponding 95% confidence intervals (95% CI) were subsequently back-transformed to the original scale for interpretation. Individual studies were weighted using the inverse-variance method.

Between-study heterogeneity was assessed using Cochran’s Q test (with statistical significance set at *p* < 0.05) and quantified using the I^2^ and τ^2^ statistics [20]. I^2^ values of 25–50%, 50–75%, and >75% were interpreted as indicating low, moderate, and high heterogeneity, respectively.

Forest plots were generated to visually present individual study estimates and the pooled prevalence. In addition, 95% prediction intervals were calculated under the random-effects framework to estimate the expected range of prevalence in future comparable studies.

To ensure the accuracy and robustness of the results, pooled estimates and heterogeneity measures were independently verified using the web-based tool Meta-Analysis Online (https://metaanalysisonline.com/; accessed 29 March 2026). This secondary platform was used exclusively for verification purposes and did not influence analytical decisions or final results.

Publication bias was not formally assessed due to the limited number of included studies, as methods to evaluate small-study effects are not considered reliable when fewer than 10 studies are available.

A sensitivity analysis was performed by sequentially excluding individual studies (leave-one-out approach) to assess the robustness of the pooled estimates.

### 2.8. Risk-of-Bias Assessment

The methodological quality of the included analytical cross-sectional studies was assessed using the Joanna Briggs Institute (JBI) Critical Appraisal Checklist for Analytical Cross-Sectional Studies [21]. This tool evaluates eight domains: (1) clarity of inclusion criteria; (2) description of study participants and setting; (3) validity and reliability of exposure measurement; (4) use of objective criteria for outcome assessment; (5) identification of confounding factors; (6) strategies to address confounding; (7) validity and reliability of outcome measurement; and (8) appropriateness of statistical analysis.

Each domain was rated as “Yes,” “No,” or “Unclear.” Studies were classified according to the number of domains rated as “No” or “Unclear” as follows: 0–2 domains were considered low risk of bias, 3–4 domains moderate risk, and ≥5 domains high risk. These thresholds were defined a priori to ensure consistency and transparency in the assessment.

The appraisal was performed independently by two reviewers (C.M.-P. and J.J.S.-E.). Any discrepancies were resolved through discussion and consensus. The results of the risk-of-bias assessment were used to inform the interpretation of the findings.

### 2.9. Analysis of GRADE Evidence Levels

The certainty of evidence for each outcome was assessed using the GRADE (Grading of Recommendations Assessment, Development and Evaluation) approach [22]. The evaluation considered the following domains: risk of bias, inconsistency, indirectness, imprecision, and publication bias (when assessable).

As all included studies were observational (cross-sectional), the certainty of evidence was initially rated as low. The certainty was subsequently downgraded or, when appropriate, upgraded based on the assessment of these domains.

Three reviewers (C.M.-P., C.S.-M., and L.C.-G.) independently performed the GRADE assessment. Discrepancies were resolved through discussion and consensus.

Summary of findings tables were constructed to present the main outcomes and corresponding certainty ratings. The final certainty of evidence for each outcome was categorized as high, moderate, low, or very low and was used to inform the interpretation of the findings.

## 3. Results

### 3.1. Study Selection

**Figure 1:**
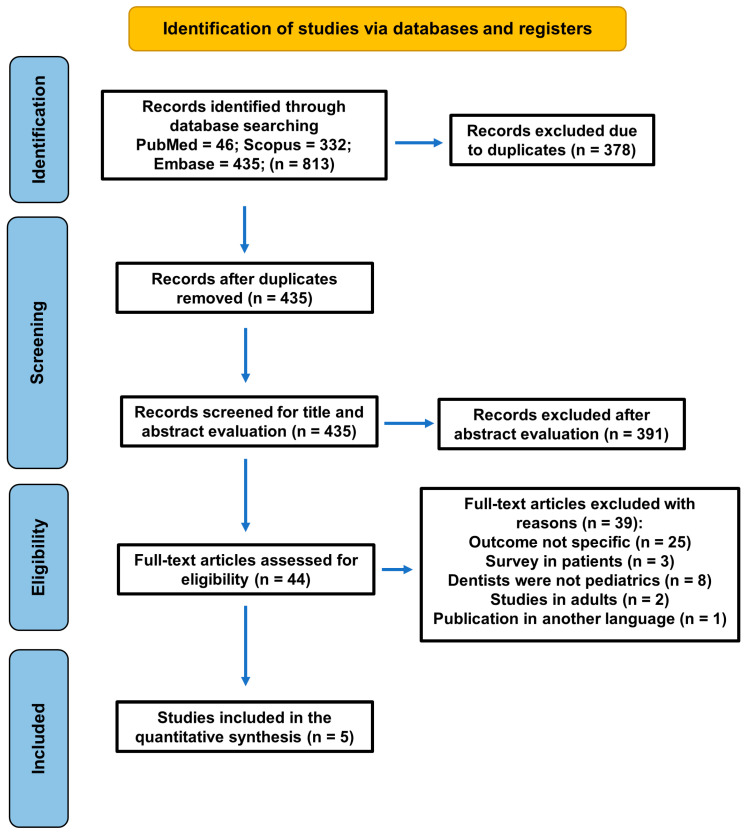
PRISMA flow diagram illustrating the study selection and screening process.

The study selection process is illustrated in Figure 1. A total of 813 records were initially identified through database searching (PubMed = 46, Scopus = 332, Embase = 435). After removal of 378 duplicates, 435 records remained for screening. Of these, 391 records were excluded based on title and abstract evaluation. The full texts of 44 articles were assessed for eligibility, of which 39 were excluded for the following reasons: not corresponding to the objective of the review (*n* = 25), survey conducted in patients (*n* = 3), inclusion of non-pediatric dentists (*n* = 8), studies focused on adults (*n* = 2), and publication in another language (*n* = 1) (Table 2). Ultimately, five studies were included in the qualitative synthesis.

### 3.2. Characteristics of the Included Studies

The main characteristics of the included studies are summarized in Table 3. All five studies were cross-sectional surveys published between 2016 [23] and 2024 [24], conducted across different geographical regions, including Europe [24,25], Asia [23,26], and the United States [27]. Sample sizes ranged from 98 [24] to 197 [27] pediatric dentists. The number of distributed questionnaires varied substantially across studies, ranging from 360 to 3434, with response rates between 20% and 78.6%. The highest response rate was reported by Ozmen and Sahin [24], whereas the lowest was observed in Vasudavan et al. [27].

Notably, there was considerable heterogeneity in the diagnostic approach to pulpitis across studies. While some investigations applied clinical diagnostic criteria based on signs and symptoms or used detailed clinical scenarios to define pulpitis, others relied on non-standardized diagnostic labels without clearly defined criteria. This variability in diagnostic definitions may have contributed to differences in reported prescribing practices and should be taken into account when interpreting the findings.

### 3.3. Pattern of Antibiotic Prescribing in Pulpitis

The patterns of antibiotic prescribing among pediatric dentists for pulpitis are summarized in Table 4. Antibiotic prescribing practices among pediatric dentists showed considerable variability across the included studies (Table 4). For pulpitis, prescribing rates ranged widely from 0.6% to 50.0%, with the highest prevalence reported by Vasudavan et al. (2019) [27]. Two studies reported statistically significant differences when compared with general dentists, indicating a more conservative prescribing approach among pediatric specialists [23,26].

In terms of antibiotic selection, amoxicillin and amoxicillin–clavulanic acid were consistently identified as the most commonly prescribed first-line agents [23,24,25,26,27]. For patients with reported penicillin allergy, clindamycin was the most frequently used alternative, although other options such as clarithromycin and combination therapies were also reported in some studies [23,24,25,26].

The duration of antibiotic therapy was relatively consistent across studies, with most prescriptions ranging from 5 to 7 days when reported [23,24,25,26].

Overall, these findings highlight substantial heterogeneity in prescribing patterns, reflecting differences in clinical decision-making and suggesting a tendency toward increased antibiotic use in certain clinical scenarios, despite the limited indication for antibiotic therapy in pulpitis [12,20].

### 3.4. Data Analysis: Meta-Analysis of Antibiotic Prescribing Patterns for Pulpitis

**Figure 2:**
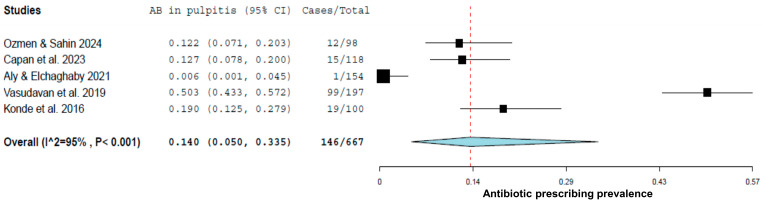
Forest plot showing the pooled prevalence of antibiotic prescribing for pulpitis among pediatric dentists. Pooled estimates were calculated using a random-effects model. Horizontal lines represent 95% confidence intervals, and squares indicate individual study estimates. The diamond represents the pooled prevalence. Heterogeneity was assessed using the I^2^ statistic.

A meta-analysis of proportions was conducted to estimate the pooled prevalence of antibiotic prescribing for pulpitis among pediatric dentists (Figure 2). Five studies comprising a total of 667 participants were included in the quantitative synthesis.

Reported antibiotic prescribing rates varied substantially across studies, ranging from 0.6% to 50.0%. Although the pooled prevalence estimated using a random-effects model was 14.0% (95% CI: 5.0–33.5%), the very high heterogeneity observed across studies limits the interpretability and generalizability of this summary estimate. Notably, one study reported a markedly higher prevalence, contributing to the dispersion of estimates. However, substantial variability was observed across individual studies, with reported prescribing rates ranging from 0.6% to 50.0%. Notably, one study reported a markedly higher prevalence, contributing to the dispersion of estimates.

Between-study heterogeneity was considerable, as indicated by a statistically significant Cochran’s Q test (*p* < 0.001) and a high I^2^ value of 95%, suggesting that most of the observed variability was due to true differences in prescribing practices rather than chance alone. The estimated between-study variance (τ^2^) further supported the presence of substantial heterogeneity.

The 95% prediction interval ranged from 0.2% to 92.2%, indicating that the prevalence of antibiotic prescribing for pulpitis may vary substantially across comparable clinical settings. This very wide interval further supports the limited generalizability of the pooled estimate and reflects the strong influence of contextual and methodological differences across studies.

Overall, although the pooled estimate suggests a relatively low average prescribing rate, the high heterogeneity underscores the inconsistency in clinical practice and highlights the persistence of inappropriate antibiotic use in certain settings, despite clear guideline recommendations discouraging antibiotic therapy for pulpitis in the absence of systemic involvement.

### 3.5. Sensitivity Analysis

**Figure 3:**
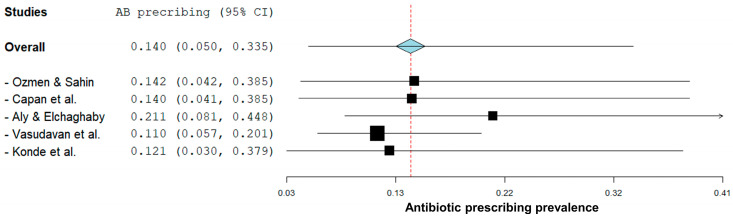
Forest plot from the sensitivity analysis of antibiotic prescribing for pulpitis among pediatric dentists. The pooled prevalence was recalculated after sequential exclusion of individual studies (leave-one-out approach). Squares represent individual study estimates, with horizontal lines indicating 95% confidence intervals, and their size proportional to study weight. The diamond represents the pooled estimate under each scenario using a random-effects model. The vertical dashed line indicates the overall pooled estimate from the primary analysis.

A sensitivity analysis was performed using a leave-one-out approach to evaluate the robustness of the pooled prevalence of antibiotic prescribing for pulpitis (Figure 3). The results showed that the overall estimate remained relatively stable across most scenarios, indicating that no single study substantially altered the direction of the findings.

However, exclusion of the study with the highest reported prescribing rate resulted in a noticeable reduction in the pooled prevalence and a partial decrease in heterogeneity. This suggests that this study had a disproportionate influence on the overall estimate, likely due to differences in study design, clinical scenarios, or prescribing context.

Despite this, the overall conclusions remained unchanged, with antibiotic prescribing for pulpitis persisting at non-negligible levels despite limited clinical indication. These findings support the robustness of the primary analysis while highlighting the impact of between-study variability on pooled estimates.

### 3.6. Risk-of-Bias Assessment

The methodological quality of the included studies was assessed using the Joanna Briggs Institute (JBI) Critical Appraisal Checklist for Analytical Cross-Sectional Studies. The detailed assessment is presented in Table 5. Overall, two studies were classified as having low risk of bias (Capan et al. [25] and Vasudavan et al. [27]), while the remaining three studies were rated as moderate risk of bias. No studies were classified as high risk.

Across studies, inclusion criteria, participant characteristics, and study settings were generally well described, and most studies applied appropriate statistical analyses. Outcome measurement was also generally adequate. However, several methodological limitations were identified. The most common sources of bias were related to inadequate identification of confounding factors and the lack of strategies to control for confounding. In addition, some studies showed unclear or insufficient reporting regarding the validity and reliability of exposure and outcome measurements.

Only two studies demonstrated a more robust methodological approach, including valid exposure assessment and appropriate strategies to address confounding variables [25,27]. In contrast, the remaining studies presented limitations in these domains, which may have affected the accuracy and comparability of the reported prescribing practices.

Overall, the predominance of studies with moderate risk of bias suggests that the findings should be interpreted with caution. These limitations were taken into account in the assessment of the overall certainty of evidence using the GRADE approach.

### 3.7. GRADE Assessment of the Certainty of Evidence

The certainty of evidence for each outcome, assessed using the GRADE approach, is summarized in Table 6. As all included studies were observational cross-sectional surveys, the certainty of evidence was initially considered low. The final certainty ratings varied according to the consistency and precision of the findings across outcomes.

The certainty of evidence for antibiotic prescribing in pulpitis was rated as low because of substantial inconsistency and imprecision, reflected by the marked variability in prescribing rates and the very high heterogeneity across studies. In contrast, evidence regarding first-line antibiotic selection and duration of therapy was considered moderate, as prescribing patterns were relatively consistent across studies and concerns regarding indirectness and imprecision were less pronounced. The certainty of evidence for antibiotic choice in penicillin-allergic patients remained low due to variability in reported prescribing practices and inconsistency in alternative regimens.

These certainty ratings were considered during interpretation of the findings and highlight the need for further standardized research in this field.

## 4. Discussion

This systematic review identified variability in antibiotic prescribing practices among pediatric dentists and revealed persistent discrepancies between clinical practice and evidence-based recommendations. Although overall prescribing rates for pulpitis were relatively low, antibiotic use remains present in clinical scenarios where it is not indicated.

Differences across studies were particularly evident in the management of pulpitis, reflecting variation in diagnostic interpretation and clinical decision-making. The absence of standardized diagnostic criteria likely contributed to these inconsistencies and may partially explain the divergence in prescribing patterns. These findings suggest that antibiotic prescribing is not always driven by clear clinical indications and may be influenced by additional contextual factors.

Regarding antibiotic selection, amoxicillin and amoxicillin–clavulanic acid were consistently reported as the preferred first-line agents, in line with their established efficacy against common odontogenic pathogens. However, the frequent use of broad-spectrum antibiotics raises concerns about potential overuse and its contribution to antimicrobial resistance [28]. Clindamycin was the most commonly reported alternative in patients with penicillin allergy, despite increasing concerns regarding its safety profile, particularly its association with Clostridioides difficile infection [12].

In contrast, the duration of antibiotic therapy was relatively consistent, with most prescriptions ranging from 5 to 7 days. However, appropriate duration does not ensure rational prescribing when the initial indication may be unjustified. In this context, the decision to prescribe antibiotics remains the primary concern.

The certainty of evidence ranged from low to moderate, primarily due to the observational design of the included studies and methodological heterogeneity. These limitations should be considered when interpreting the findings.

Importantly, the application of a random-effects model does not fully resolve the interpretative limitations associated with extreme heterogeneity (I^2^ = 95%). The observed variability across studies appears to reflect true contextual and methodological differences rather than random variation alone. Therefore, the pooled prevalence estimate should be interpreted cautiously, and greater emphasis should be placed on the wide range of observed prescribing rates across studies (0.6–50.0%).

### 4.1. Consistency of the Findings

The findings of this review are consistent with previous international evidence indicating that inappropriate antibiotic prescribing in dentistry remains a persistent global concern [8,19,29]. In particular, pulpitis has been repeatedly identified as a condition frequently associated with unjustified antibiotic use, despite clear recommendations supporting operative treatment as the primary management approach [20,29].

Several factors described in the literature may help explain these patterns. Diagnostic uncertainty, misconceptions regarding the role of antibiotics in pain and inflammation control, and non-clinical influences—such as patient expectations—have all been associated with inappropriate prescribing behavior. In addition, the lack of standardized diagnostic criteria observed across studies may contribute to variability in clinical decision-making and inconsistencies in reported prescribing practices.

The lower prescribing rates observed among pediatric dental specialists compared with general dentists are in line with previous evidence suggesting that advanced training is associated with more appropriate antibiotic use. Greater diagnostic confidence and stronger adherence to guideline-based management may explain these differences. However, inappropriate prescribing persists even among specialists, indicating that the gap between evidence and clinical practice remains unresolved [21,22,30]. Similarly, Delgado-Giugni et al. [31] reported higher prescribing rates among general dentists compared with endodontists, reinforcing the role of professional training in prescribing behavior.

Patterns of antibiotic selection were also consistent with existing literature, with amoxicillin and amoxicillin–clavulanic acid as the most commonly prescribed first-line agents. Nevertheless, the frequent use of broad-spectrum antibiotics may be excessive in certain clinical scenarios and may contribute unnecessarily to antimicrobial resistance [32]. Clindamycin was the most frequently reported alternative in patients with penicillin allergy, despite increasing concerns regarding its safety profile, particularly its association with Clostridioides difficile infection [13].

Although treatment duration was generally consistent with current recommendations, this apparent alignment should be interpreted cautiously, as appropriate duration does not compensate for inappropriate indication. The decision to prescribe antibiotics remains the primary concern.

These findings are particularly relevant in the context of antimicrobial resistance, a major global public health challenge. The World Health Organization has emphasized the urgent need to reduce unnecessary antibiotic use across healthcare settings [33]. Given that most dental pain in pediatric patients is associated with localized inflammatory conditions rather than systemic infection [34], continued prescribing in these cases represents avoidable antibiotic exposure and a key target for antimicrobial stewardship interventions [35].

### 4.2. Clinical Implications

The findings of this systematic review have important implications for clinical practice in pediatric dentistry. Despite the availability of clear evidence-based recommendations, inappropriate antibiotic prescribing remains frequent, particularly in clinical scenarios such as pulpitis, where local operative treatment is sufficient and systemic antibiotics are not indicated [12,13,20].

These results highlight the need to prioritize accurate diagnosis and timely operative management as the cornerstone of treatment for pulpitis. The very wide prediction interval reported in the present review further emphasizes the substantial variability in antibiotic prescribing practices across clinical settings. This finding supports the interpretation that contextual and methodological differences between studies strongly influence prescribing behavior and limits the generalizability of the pooled prevalence estimate.

In most cases, effective local interventions—such as pulpotomy, pulpectomy, or extraction—can resolve the underlying pathology without the need for antibiotic therapy [12,14,15]. Therefore, strengthening diagnostic skills and clinical decision-making should be a central focus in both undergraduate education and continuing professional development [28,36].

The frequent use of broad-spectrum antibiotics, particularly amoxicillin–clavulanic acid, suggests a tendency toward overtreatment in situations where narrower-spectrum agents may be sufficient when antibiotics are truly indicated. This practice may contribute unnecessarily to antimicrobial resistance and should be carefully reconsidered [32]. Similarly, the widespread use of clindamycin as an alternative in patients with reported penicillin allergy warrants critical evaluation, given its association with adverse outcomes such as Clostridioides difficile infection and recent recommendations discouraging its routine use as a first-line alternative [12,13].

Importantly, this review indicates that the main challenge is not the duration of antibiotic therapy, which appears generally consistent with current recommendations, but rather the appropriateness of the indication for prescribing. This is consistent with previous evidence suggesting that inappropriate initiation of antibiotics represents the primary driver of misuse in dental practice [19,29]. This underscores the need for antimicrobial stewardship strategies that focus on improving clinical judgment at the point of care, rather than solely on optimizing dosing regimens.

In this context, targeted interventions such as clinical decision-support tools, case-based guideline dissemination, and structured educational programs may help bridge the gap between evidence and practice [28,36,37]. Additionally, improving communication with parents and caregivers is essential, as their expectations may influence prescribing behavior [38]. Clear explanations regarding the limited role of antibiotics in managing dental pain and localized infections can reduce pressure on clinicians and support more rational prescribing.

From a broader perspective, optimizing antibiotic use in pediatric dentistry requires a multifaceted approach that integrates clinician education, guideline implementation, and system-level support. Reducing unnecessary antibiotic exposure in children is a key priority not only to limit antimicrobial resistance, but also to minimize avoidable adverse effects and improve overall patient safety [8,33].

Future research should focus on evaluating the effectiveness of targeted antimicrobial stewardship interventions specifically designed for pediatric dental settings.

### 4.3. Factors Explaining the Variability

Multiple contextual factors may account for the substantial variability identified across studies. One key explanation is diagnostic uncertainty, which has consistently been recognized as a barrier to appropriate antibiotic use and may lead clinicians to prescribe defensively. This may be influenced by inconsistent diagnostic criteria, limited access to diagnostic tools such as pulp vitality testing, and differences in clinicians’ confidence when distinguishing between pulpal conditions requiring operative treatment rather than antibiotics [8].

Another possible explanation is restricted access to urgent dental care. In settings where definitive procedures such as pulpotomy or pulpectomy cannot be performed promptly, antibiotics may be inappropriately used as a temporary substitute for operative intervention. Differences in professional training may also contribute to this heterogeneity. The contrast observed between general dental practitioners and pediatric dentists is consistent with previous evidence suggesting that advanced training is associated with reduced antibiotic prescribing, likely due to improved diagnostic reasoning, greater familiarity with emergency procedures, and stronger adherence to evidence-based recommendations [29].

Parental expectations may represent an additional driver of prescribing variability. Antibiotics are often perceived by caregivers as a means of relieving pain or accelerating recovery, which may place pressure on clinicians to prescribe even when not indicated. This influence may be greater in healthcare systems where patient satisfaction affects clinical decisions or where communication barriers make it more difficult to explain why antibiotics may be unnecessary [38].

Broader health system and sociocultural factors may also play an important role. International differences in regulation, stewardship implementation, and professional prescribing autonomy have been linked to variation in antibiotic use. Likewise, disparities in the dissemination and enforcement of national guidelines, together with cultural norms surrounding antibiotic use and self-medication, may further contribute to heterogeneous prescribing patterns.

The study by Vasudavan et al. differed substantially from the remaining studies in several methodological and contextual aspects. First, it employed detailed clinical scenarios involving pulpitis-related presentations, which may have increased the likelihood of antibiotic prescribing compared with studies based on general diagnostic labels. Second, the study was conducted in the United States, where healthcare organization, access to emergency dental care, and medico-legal considerations may influence prescribing behavior differently from other regions. Finally, differences in diagnostic criteria and clinical interpretation of pulpitis likely contributed to the markedly higher prevalence observed. Importantly, the persistence of substantial heterogeneity after exclusion of this study suggests that the variability across studies is structural and multifactorial rather than driven by a single outlier.

Collectively, these findings suggest that inappropriate antibiotic use in pulpitis is multifactorial and shaped by a complex interaction of clinical, systemic, and sociocultural influences. The sensitivity analysis further underscores the influence of contextual and methodological differences across studies, which should be considered when interpreting pooled estimates.

### 4.4. Strengths and Limitations

This systematic review has several methodological strengths that enhance the robustness and relevance of its findings. The study was designed and reported in accordance with the PRISMA 2020 guidelines (Supplementary Materials) and was prospectively registered in PROSPERO, which improves transparency and reduces the risk of reporting bias. The use of the CoCoPop framework allowed for a structured and appropriate formulation of the research question, particularly suited to observational studies addressing patterns of clinical practice. In addition, the comprehensive search strategy across multiple databases increased the likelihood of identifying relevant studies.

The methodological quality of the included studies was systematically assessed using a validated tool (JBI), and the certainty of evidence was evaluated using the GRADE approach, providing a transparent assessment of the strength of the findings. Furthermore, the inclusion of a meta-analysis of proportions allowed for the quantitative synthesis of prescribing patterns and facilitated the exploration of variability across studies.

However, several limitations should be acknowledged. All included studies were cross-sectional in design, which limits causal inference and may be subject to reporting bias. In addition, most data were derived from self-reported questionnaires, which may not accurately reflect actual prescribing behavior and may lead to overestimation of adherence to clinical guidelines.

Substantial heterogeneity was observed across studies, likely due to differences in study populations, geographic settings, survey design, and, importantly, the lack of standardized diagnostic criteria for pulpitis and related conditions. This variability may have affected the comparability of results and the precision of pooled estimates. Although a random-effects model was applied to account for between-study variability, the high level of heterogeneity reduces confidence in the generalizability of the findings.

The relatively small number of included studies also represents a limitation, restricting the ability to perform subgroup analyses or formally assess publication bias. In addition, the restriction to English-language publications may have introduced language bias, and relevant studies published in other languages may have been missed.

Despite these limitations, the consistency of several key findings—particularly regarding antibiotic selection and the persistence of inappropriate prescribing—supports the validity of the overall conclusions. Nevertheless, the results should be interpreted with caution, and further high-quality, standardized research is needed to better characterize antibiotic prescribing practices and to evaluate the effectiveness of antimicrobial stewardship interventions in pediatric dentistry.

## 5. Conclusions

Pediatric dentists appear to adopt a more conservative approach to antibiotic prescribing than general practitioners; however, prescribing practices remain variable and are not consistently aligned with current clinical guidelines. This is particularly evident in conditions such as pulpitis, where antibiotic therapy is generally not indicated and local operative management is sufficient in the absence of systemic involvement.

Amoxicillin and amoxicillin–clavulanic acid were the most commonly prescribed antibiotics, while clindamycin remained a frequent alternative in patients with reported penicillin allergy, despite growing concerns regarding its safety profile.

The overall certainty of evidence ranged from low to moderate, reflecting the methodological limitations and heterogeneity of the included studies. These findings highlight a persistent gap between evidence-based recommendations and clinical practice. Moreover, the substantial variability across studies suggests that prescribing practices are strongly influenced by contextual and methodological differences.

Strengthening adherence to clinical guidelines, improving diagnostic accuracy, and promoting targeted antimicrobial stewardship strategies should be prioritized to reduce unnecessary antibiotic use in pediatric dental care. Further high-quality research is needed to better understand prescribing behaviors and to evaluate interventions aimed at optimizing antibiotic use in this field. Reducing unnecessary antibiotic use in pediatric dentistry represents a critical opportunity to contribute to global antimicrobial stewardship efforts.

## Figures and Tables

**Table 1:** Literature search in databases.

Database	Exact Search String Used	No. of Articles	Date of Last Search
PubMed/ MEDLINE	((“Pulpitis”[MeSH Terms]) AND (“Anti-Bacterial Agents”[MeSH Terms] OR antibiotic*[Title/Abstract] OR antimicrobial*[Title/Abstract]))	46	From 2016 to 15 March 2026
Scopus	TITLE-ABS-KEY(“pulpitis”) AND TITLE-ABS-KEY(antibiotic* OR antimicrobial*)	332	From 2016 to 15 March 2026
EMBASE	(pulpitis/exp) AND (‘antibiotic agent’/exp OR antibiotic*:ti,ab OR antimicrobial*:ti,ab)	435	From 2016 to 15 March 2026

**Table 2:** Excluded studies and their reasons for exclusion.

Reasons	Excluded Studies
Does not correspond with the objective of this review	Roopan et al. 2026 Sobhy et al. 2026 Shetty et al. 2025 Xie et al. 2025 Yi et al. 2025 Mohn et al. 2025 Phan et al. 2025 Huang et al. 2024 Dimri et al. 2024 Pekpinarli et al. 2024 Xie et al. 2024 Khijmatgar et al. 2024 Boreak et al. 2024 Xie et al. 2023 Saade et al. 2023 Colombo et al. 2022 Seewald et al. 2021 Zanjir et al. 2020 Tampi et al. 2019 Pourhajibagher and Bahador 2019 Wu et al. 2019 Martins et al. 2018 Moon et al. 2018 da Silva et al. 2018 Lang et al.2016
Survey in patients	Edwards et al. 2024 Ilyas et al. 2021 Dana et al. 2019
Dentists were not pediatric	Kalantzis et al. 2024 Karobari et al. 2021 Gemmell et al. 2020 Agnihotry et al. 2019 Agnihotry et al. 2019 Bolfoni et al. 2018 Germack et al. 2017 Segura-Egea et al. 2017
Studies with adults	Yaqoob et al. 2024 Martín-Jiménez et al. 2018
In another language	Agustí 2019

**Table 3:** Characteristics of the included studies.

Authors & Year	Country	Study Type	Distributed Questionnaires	Response Rate (%)	Diagnosis of Pulpitis
Ozmen and Sahin 2024 [24]	Turkey	Cross-sectional survey	360	78.6	Clinical diagnostic criteria through signs and symptoms
Capan et al. 2023 [25]	Turkey	Cross-sectional survey	2100	24.2	Diagnosis reported as a label without defined clinical criteria
Aly and Elchaghaby 2021 [26]	Egypt	Cross-sectional survey	1512	25.0	Diagnosis reported as a label without defined clinical criteria
Vasudavan et al. 2019 [27]	USA	Cross-sectional survey	3434	20.0	Clinical scenarios containing complete criteria for pulpitis
Konde et al. 2016 [23]	India	Cross-sectional survey	NR	NR	Diagnosis reported as a label without defined clinical criteria

NR: not reported.

**Table 4:** Pattern of antibiotic prescribing for pulpitis among pediatric dentists.

Authors & Year	Respondents	AB Prescription in Pulpitis *n*, (%)	First-Line Antibiotic	Alternative Antibiotic (Penicillin-Allergic)	Duration (Days)
Ozmen and Sahin 2024 [24]	98	12 (12.5)	Amoxicillin–clavulanic acid	Clarithromycin	7 days
Capan et al. 2023 [25]	118	15 (12.8)	Amoxicillin–clavulanic acid	Clindamycin	5–7 days
Aly and Elchaghaby 2021 [26]	154	1 (0.6) *	Amoxicillin–clavulanic acid	Clindamycin	5–7 days
Vasudavan et al. 2019 [27]	197	99 (50.0)	Amoxicillin	Clindamycin	NR
Konde et al. 2016 [23]	100	19 (19.0) *	Amoxicillin	Ofloxacin with ornidazole	5 days

* Statistically significant compared with general dentists; NR: not reported.

**Table 5:** Risk-of-bias assessment.

Study	Inclusion Criteria	Participants and Setting	Exposure Validity	Outcome Measurement	Confounders	Confounding Control	Outcome Validity	Statistical Analysis	No/Unclear Domains (*n*)	Risk of Bias
Ozmen and Sahin [24]	Yes	Yes	? Unclear	Yes	No	No	Yes	Unclear	4	Moderate
Capan et al. [25]	Yes	Yes	Yes	Yes	Yes	No	Yes	Yes	1	Low
Aly and Elchaghaby [26]	Yes	Yes	? Unclear	Yes	No	No	? Unclear	Yes	4	Moderate
Vasudavan et al. [27]	Yes	Yes	Yes	Yes	Yes	Yes	Yes	Yes	0	Low
Konde et al. [23]	Yes	Yes	? Unclear	Yes	No	No	? Unclear	Yes	4	Moderate

Abbreviations: Domain names correspond to the Joanna Briggs Institute Critical Appraisal Checklist for Analytical Cross-Sectional Studies.

**Table 6:** Summary of findings (GRADE assessment of certainty of evidence).

Outcome	No. of Studies/Design	Certainty of Evidence (GRADE)	Summary of Findings
Antibiotic prescribing for pulpitis	5 cross-sectional studies	LOW	Antibiotic prescribing rates for pulpitis varied substantially across studies (0.6–50.0%), with very high heterogeneity (I^2^ = 95%). The pooled prevalence was 14.0% (95% CI: 5.0–33.5%), although the wide prediction interval (0.2–92.2%) limits the generalizability of the estimate.
First-line antibiotic choice	5 cross-sectional studies	MODERATE	Amoxicillin and amoxicillin–clavulanic acid were consistently reported as the most commonly prescribed first-line antibiotics across studies.
Antibiotic choice in penicillin-allergic patients	5 cross-sectional studies	LOW	Clindamycin was the most frequently reported alternative antibiotic, although variability across studies was observed, with clarithromycin and ofloxacin-based combinations also reported.
Duration of antibiotic therapy	4 cross-sectional studies	MODERATE	Most studies reported antibiotic treatment durations ranging from 5 to 7 days, showing relatively consistent prescribing patterns regarding duration.

Abbreviations: CI, confidence interval.

## Data Availability

No new data were created or analyzed in this study. Data sharing is not applicable to this article.

## Supplementary Materials

The following supporting information can be downloaded at: https://www.mdpi.com/article/10.3390/antibiotics15060586/s1. PRISMA 2020 Checklist.
